# Nomadic Enhancers: Tissue-Specific *cis*-Regulatory Elements of *yellow* Have Divergent Genomic Positions among *Drosophila* Species

**DOI:** 10.1371/journal.pgen.1001222

**Published:** 2010-11-24

**Authors:** Gizem Kalay, Patricia J. Wittkopp

**Affiliations:** 1 Department of Molecular, Cellular, and Developmental Biology, University of Michigan, Ann Arbor, Michigan, United States of America; 2 Department of Ecology and Evolutionary Biology, University of Michigan, Ann Arbor, Michigan, United States of America; University of California Davis, United States of America

## Abstract

*cis*-regulatory DNA sequences known as enhancers control gene expression in space and time. They are central to metazoan development and are often responsible for changes in gene regulation that contribute to phenotypic evolution. Here, we examine the sequence, function, and genomic location of enhancers controlling tissue- and cell-type specific expression of the *yellow* gene in six Drosophila species. *yellow* is required for the production of dark pigment, and its expression has evolved largely in concert with divergent pigment patterns. Using *Drosophila melanogaster* as a transgenic host, we examined the expression of reporter genes in which either 5′ intergenic or intronic sequences of *yellow* from each species controlled the expression of Green Fluorescent Protein. Surprisingly, we found that sequences controlling expression in the wing veins, as well as sequences controlling expression in epidermal cells of the abdomen, thorax, and wing, were located in different genomic regions in different species. By contrast, sequences controlling expression in bristle-associated cells were located in the intron of all species. Differences in the precise pattern of spatial expression within the developing epidermis of *D. melanogaster* transformants usually correlated with adult pigmentation in the species from which the *cis*-regulatory sequences were derived, which is consistent with *cis*-regulatory evolution affecting *yellow* expression playing a central role in Drosophila pigmentation divergence. Sequence comparisons among species favored a model in which sequential nucleotide substitutions were responsible for the observed changes in *cis*-regulatory architecture. Taken together, these data demonstrate frequent changes in *yellow cis*-regulatory architecture among Drosophila species. Similar analyses of other genes, combining *in vivo* functional tests of enhancer activity with *in silico* comparative genomics, are needed to determine whether the pattern of regulatory evolution we observed for *yellow* is characteristic of genes with rapidly evolving expression patterns.

## Author Summary

In order for a gene to be active, it must be turned on, or “expressed.” Instructions determining when, where, and how much a gene will be expressed are encoded by DNA sequences known as enhancers. The precise DNA sequence of a particular enhancer changes over evolutionary time, which may or may not change its effects on gene expression. Many genes are controlled by multiple enhancers and prior work has shown that the location of these enhancers within the genome tends to remain stable for long periods of evolutionary time. Here, we examine the enhancers controlling expression of a gene (*yellow*) involved in generating pigmentation diversity among fruit fly (Drosophila) species. Surprisingly, we find that not only have the sequence and function of individual enhancers changed among Drosophila species, but so has the location of these enhancers within the genome of each species. This finding is important because it demonstrates a type of evolutionary change affecting DNA sequence elements critical for gene expression that is currently under appreciated and should be considered when searching for enhancers in related species.

## Introduction

The production of a complex, multi-cellular organism requires transcription of a subset of the genome in each cell. This process, known as gene expression, is controlled by *cis*-regulatory DNA sequences that interact with *trans*-regulatory proteins and RNAs. These *cis*-regulatory sequences include “enhancers”, which contain binding sites for transcription factors. The specific combination of transcription factor binding sites within an enhancer determines its activity and specifies the timing, location, and abundance of expression for the gene it regulates. Many genes, especially those involved in development, are controlled by multiple enhancers, each of which controls a subset of the gene's total expression pattern and can be located 5′, 3′ or in an intron of the gene whose transcription it regulates. Like all DNA, *cis*-regulatory sequences are subject to the unavoidable process of mutation, which – over evolutionary time – can change enhancer sequence, enhancer function, and the genomic location of enhancers relative to the gene whose expression they control.

Comparing the *cis*-regulatory architecture of orthologous genes among species reveals how they evolve as well as which features are essential for their activity. Conserved sequences between orthologous enhancers represent putatively functional elements (e.g., [1], [2]), but conservation of DNA sequence is not strictly required for conservation of enhancer function: transcription factor binding sites are often degenerate and comparable enhancer functions can be produced by multiple arrangements of these sites [3]–[6]. Compared to enhancer sequence, enhancer location within the genome (relative to exonic sequences of the associated gene) appears to be more constrained. For example, the location of enhancers is conserved for the *even-skipped* gene between Drosophila and Sepsid species [5], which diverged over 100 million years ago, and for six Dorsal target genes between Drosophila and Anopheles or Tribolium [7], which diverged over 200 million years ago. In fact, conservation of enhancer location within the genome is something that many researchers rely upon in their search for orthologous enhancers.

Here, we investigate the evolution of *cis*-regulatory architecture controlling expression of the Drosophila *yellow* gene. Yellow is required for the production of dark melanic pigment in insects [8]–[10], and its expression during late pupal stages has evolved in a manner that often correlates with the distribution of melanins in adults [11]–[13]. In *D. melanogaster*, *yellow* expression is controlled by multiple tissue-specific enhancers, with enhancers driving expression in the pupal wing, abdomen, and thorax located 5′ of the *yellow* gene and an enhancer driving expression in bristle-associated cells located within its lone intron [12], [14]–[16]. Comparisons of *yellow* expression and regulation among species suggest that changes in *cis*-regulatory activity are most often responsible for divergent *yellow* expression patterns [11]–[14], [17], although changes in *trans*-regulatory factors also contribute to expression divergence in some species [12], [17]). Changes in the spatial pattern of *yellow* expression within the developing abdomen result from changes in orthologous enhancers located in the 5′ intergenic sequences of *yellow* [12], [14], and convergent *yellow* expression in “spots” on the developing wing results from enhancers that evolved in the 5′ intergenic region of one species and in the intron of another [11], [13], [17].

To examine the evolution of *yellow cis-*regulatory architecture more comprehensively and systematically, we determined the enhancer activity of sequences 5′ of *yellow* and in its intron for six species spanning the phylogenetic tree of the genus Drosophila. These species include members of both the *Drosophila* (*D. mojavensis, D. virilis*, and *D. grimshawi*) and Sophophora (*D. melanogaster, D. pseudoobscura*, and *D. willistoni*) subgenera and have pairwise divergence times ranging from approximately 20 to 40 million years ago [18], [19]. Surprisingly, we found that the location of *yellow* enhancer activity controlling expression in a particular tissue- or cell-type differed frequently among species, with only the enhancer controlling bristle-associated expression located in the same genomic region of all species. These differences in *cis*-regulatory architecture were accompanied by differences in enhancer activity that often correlated with species-specific pigment patterns, as expected based on prior studies [11]–[14], [17]. Sequence comparisons between pairs of species showed no clear evidence of duplications or transpositions near *yellow*, suggesting that differences in enhancer location among species evolved by sequential sequence substitutions, one or a few nucleotides at a time. To the best of our knowledge, such extensive and rapid turnover in the genomic location of enhancers has not been observed for any other eukaryotic gene.

## Results

**Figure 1:**
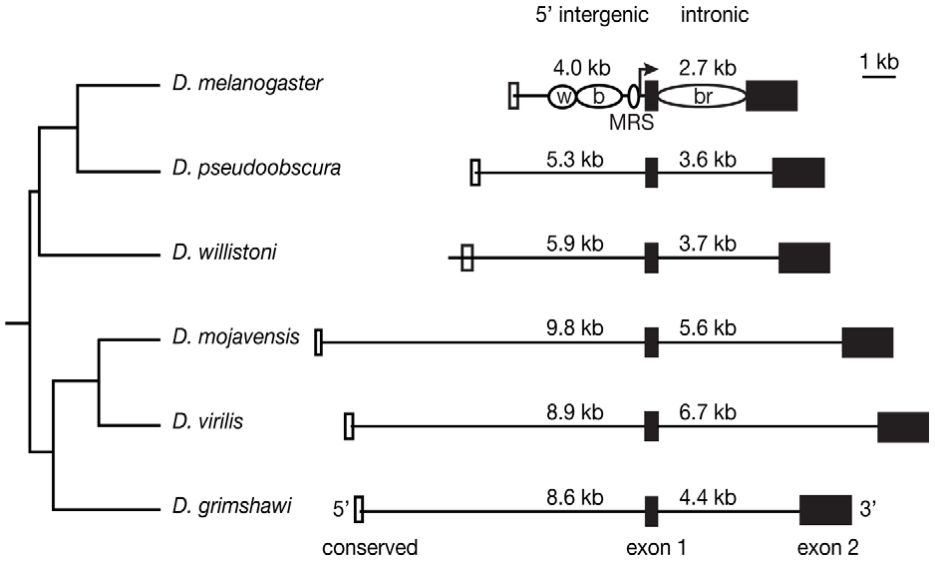
DNA sequences tested for enhancer activity vary in length among species. The size of each 5′ intergenic and intronic region tested, which ended and began, respectively at exon 1, is shown in kilobases (kb). Filled black boxes indicate exons, whereas open boxes indicate the region of conserved sequence shown in Figure S1. The black lines indicate the DNA included in each construct. Note that only *D. willistoni* includes the entire conserved 5′ block. Phylogenetic relationships among Drosophila species are indicated on the left. For *D. melanogaster*, the location of previously identified enhancers that drive expression in the wing (w), body (b) and bristles (br) of adult flies [12], [14]–[16] as well as sequences known to influence male mating success (MRS, [39]) are indicated with open ovals. Sequences have also been identified in this species that are necessary for *yellow* expression in the larval mouthparts, larval denticle belts, microsatae, tarsal claws, aristae and sex combs [15], [16], but are not shown.

To determine the *cis*-regulatory architecture of *yellow* in each of six *Drosophila* species, we constructed reporter genes that used species-specific 5′ intergenic or intronic sequences of *yellow* to drive expression of a nuclear Green Fluorescent Protein (GFP) in transgenic *D. melanogaster*. The 5′ intergenic regions surveyed began near a highly-conserved region of sequence (Figure S1) located 5′ of the previously characterized wing and body enhancers of *D. melanogaster yellow* [12], [14]–[17] and extended 3′ to the beginning of the first exon of *yellow* (Figure 1). This region includes all of the 5′ intergenic DNA contained within *yellow* transgenes that fully rescue *yellow* null mutant phenotypes in *D. melanogaster* [15] and *D. virilis* [12], suggesting that these constructs are likely to contain all 5′ enhancers affecting *yellow* expression. The intronic constructs began and ended with sequences in the first and second exons, respectively. DNA fragments tested ranged from 4 to 9.8 kb for the 5′ intergenic regions and from 2.7 to 6.7 kb for the intronic regions (Figure 1). Each of the twelve reporter genes was independently integrated into the same pre-determined location of the *D. melanogaster* genome using the phiC31 integrase system [20], and expression of the GFP reporter gene in transgenic pupae 70–80 hours after puparium formation was examined by confocal microscopy with identical settings for all samples. A reporter gene lacking putative enhancer sequences was also inserted into the same genomic location and used to determine background levels GFP expression.

### Genomic location of tissue-specific enhancers differs among species

**Figure 2:**
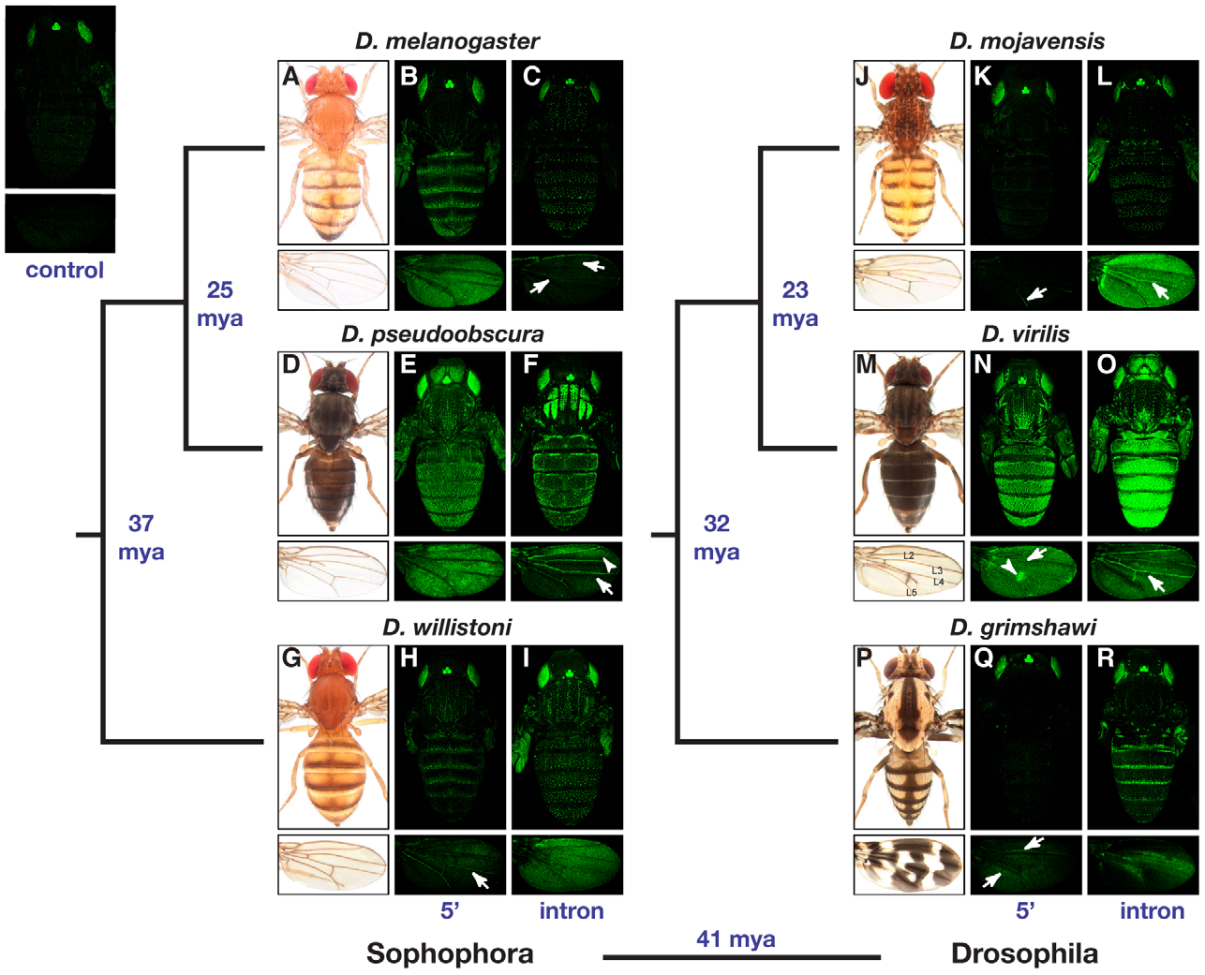
Location and activity of the *yellow* body and wing enhancers is highly divergent among Drosophila species. Expression (shown in green) of nuclear Green Fluorescent Protein (GFP) activated in transgenic *D. melanogaster* by the 5′ intergenic (5′) and intronic (intron) fragments of DNA shown in Figure 1 from the six species indicated is shown. For each species, the panel of six images includes pictures of the dorsal side of the body (top) and wings (bottom). From left to right, panels show an adult specimen of the species indicated (images provided by N. Gompel), a transgenic *D. melanogaste*r pupa carrying the corresponding 5′ intergenic sequence-GFP reporter gene, and a transgenic *D. melanogaste*r pupa carrying the corresponding intronic sequence-GFP reporter gene. Expression patterns indicated with arrows and arrowheads are described in the main text. Divergence times [17], [18] between lineages are shown in blue in millions of years ago (mya). Fluorescence observed in the body (top) and wing (bottom) of a *D. melanogaster* pupa carrying the GFP reporter gene without any putative *yellow* enhancer sequences cloned upstream is shown in the top left of the figure, and serves as a negative control. In each case, the GFP-expressing image shown is from female pupae, 70–80 hours old, and is representative of the at least 10 individual specimens examined of each genotype. Note that bright GFP expression in eyes and ocelli (located between eyes on each head) in all images, including the control, is activated by the transformation marker gene and not the y*ellow* 5′ intergenic or intronic sequences.

All DNA fragments tested for enhancer activity were sufficient to activate GFP expression above background levels in at least one tissue during the pupal stage examined (Figure 2). A DNA fragment was considered to lack enhancer activity in a particular tissue if it failed to drive GFP expression above background in that tissue. Reporter genes containing 5′ intergenic and intronic sequences from *D. melanogaster* drove expression patterns consistent with prior studies [12], [14]–[17]: the 5′ intergenic sequence drove expression in the epidermal cells of the abdomen, thorax and wing (Figure 2B), whereas the intronic sequence drove expression in bristle-associated cells (Figure 2C). We also observed faint expression in wing veins activated by the *D. melanogaster* intronic sequence (Figure 2C, arrows) – an enhancer activity that (to the best of our knowledge) has not previously been reported in *D. melanogaster*. Reporter gene expression was similarly used to infer the location of tissue- and cell-type specific enhancers in each of the other five species. Locations for enhancers that drive expression in the epidermal cells of the abdomen, thorax, wing, and head; in the wing veins; and in bristle-associated cells are summarized in the following paragraphs.

For each species, enhancers driving expression in epidermal cells of the abdomen, thorax, wing, and (when expression was present) head were typically found in the same genomic region; however, the location of this region differed among species and half of the species showed evidence of epidermal cell enhancers in both the 5′ intergenic and intronic regions. Enhancers driving expression in epidermal cells of the abdomen, thorax, and wing were observed in the 5′ intergenic regions of all three Sophophora species (i.e., *D. melanogaster*, *D. pseudoobscura*, and *D. willistoni*) and *D. virilis* from the Drosophila subgenus (Figure 2B, 2E, 2H, 2N) as well as in the introns of *D. pseudoobscura* and all three species from the Drosophila subgenus (i.e., *D. mojavensis, D. virilis*, and *D. grimshawi*) (Figure 2F, 2L, 2O, and 2R). In addition, the intron from *D. willistoni* drove expression in the epidermal cells of the thorax and wing (Figure 2I), and the *D. grimshawi* 5′ intergenic region drove expression in a small region of epidermal cells flanking two of the wing veins (Figure 2Q, arrows). Expression in head epidermal cells was observed only in *D. pseudoobscura* and *D. virilis*, with the enhancer controlling this expression located in the 5′ intergenic or intronic regions of these species, respectively (Figure 2E and 2O).

The genomic location of enhancers driving expression in wing veins was also variable among species. In the subgenus Sophophora, the two most closely related species, *D. melanogaster* and *D. pseudoobscura*, both showed this enhancer activity in the intron (Figure 2C and 2F, arrows), whereas the more distantly related *D. willistoni* showed wing vein enhancer activity in the 5′ intergenic sequence (Figure 2H, arrow). In the subgenus Drosophila, both 5′ intergenic and intronic sequences from *D. mojavensis* and *D. virilis* drove expression in the wing veins (Figure 2K, 2L, 2N, and 2O, arrows), but no wing vein expression was observed from either reporter gene containing *D. grimshawi* sequence (Figure 2Q and 2R).

Expression in bristle-associated cells of both the body and wing was controlled by intronic sequences from all six species, making it the only *yellow* enhancer activity whose genomic location appears to be conserved within the genus Drosophila (Figure 2C, 2F, 2I, 2L, 2O, and 2R).

### Divergent activity of *yellow* enhancers often correlates with divergent pigmentation

The spatial patterns of reporter gene expression in epidermal cells of the abdomen, thorax, and (less frequently) wing often differed between species (Figure 2). With few exceptions (noted below), sequences from each species activated GFP expression in transgenic *D. melanogaster* hosts in patterns that correlated with adult pigmentation of the species from which the enhancer sequences were derived. In the abdomen, for example, *D. melanogaster, D. willistoni*, and *D. grimshawi* all have dark stripes at the posterior edge of each dorsal abdominal segment (Figure 2A, 2G, and 2P) and show similar stripes of reporter gene expression in each abdominal segment driven by either their 5′ intergenic or intronic sequences (Figure 2B, 2H, and 2R). *D. mojavensis*, however, also has pigment stripes on its dorsal abdomen, but the weak abdominal reporter gene expression observed was not restricted to these stripes (Figure 2L). In addition, *D. mojavensis* has a series of pigment spots on its head and thorax (Figure 2J), and *D. grimshawi* has dark pigments along the dorsal midline in the abdomen and in the thorax (Figure 2P), neither of which are reflected in the expression patterns of the corresponding species-specific reporter genes (Figure 2K, 2L, 2Q, and 2R). Finally, *D. pseudoobscura* and *D. virilis* have an overall dark body color and faint stripes on the thorax (Figure 2D and 2M), all of which are reflected in the reporter gene expression patterns for both species (Figure 2E, 2F, 2N, and 2O).

Partial correlations between reporter gene expression and adult pigmentation were also seen in the wing. *D. virilis* has a visible spot of dark pigment surrounding one of its cross-veins (Figure 2M), and *D. grimshawi* has an elaborate pattern of pigment spots (Figure 2P). The 5′ intergenic region from *D. virilis* drove higher levels of expression in cells that will give rise to the pigmented spot surrounding L4-L5 cross-vein than in the rest of the wing (Figure 2N, arrowhead), whereas the *D. grimshawi* intron drove elevated expression in a subset of wing epidermal cells in a pattern that did not correlate well with adult *D. grimshawi* wing pigmentation (Figure 2R). Interestingly, the *D. pseudoobscura* intron drove elevated expression in an anterior spot of the wing (Figure 2F, arrowhead) despite the fact that *D. pseudoobscura* lacks any obvious dark pigment patterns in this region.

### Changing *cis*-regulatory architecture: moving existing elements or *de novo* construction and destruction?

As described above, similar tissue-specific enhancer activities were found in different genomic regions among the species surveyed. Such changes in *cis*-regulatory architecture can be achieved through (1) the movement of existing enhancers via duplications and/or transpositions of DNA sequence or (2) the *de novo* construction or destruction of transcription factor binding sites individually via sequential nucleotide changes. Each of these mechanisms is expected to produce a different pattern of sequence similarity between species. For example, consider *D. melanogaster*, which has an enhancer driving expression in abdominal epidermal cells in its 5′ intergenic region (Figure 2B), and *D. pseudoobscura*, which has two enhancers driving expression in abdominal epidermal cells located in its 5′ intergenic and intronic regions (Figure 2E and 2F). If the intronic enhancer in *D. pseudoobscura* resulted from a duplication of the 5′ enhancer shared with *D. melanogaster*, sequence similarity is expected between the 5′ region of *D. melanogaster* and the intron of *D. pseudoobscura* as well as between the 5′ intergenic and intronic sequences of *D. pseudoobscura* itself. If, however, a more gradual sequence substitution process caused either the loss of abdominal epidermal cell enhancer activity in the *D. melanogaster* intron or the gain of this activity in the *D. pseudoobscura* intron, regions of sequence similarity are expected to be collinear between species. That is, the introns of both species should share greater sequence similarity with each other than either does with the other species' 5′ intergenic sequence and vice versa.

**Figure 3:**
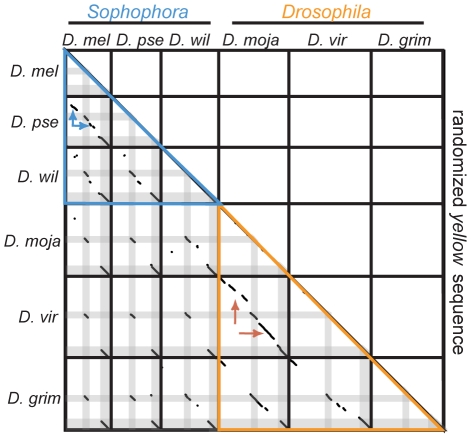
*yellow* sequences show no evidence of large duplications or transpositions. Pairwise comparisons of *yellow* genes and their associated 5′ intergenic regions from each species to each other species (and to themselves) are shown in the lower left, and a comparison of each species' sequence to a randomized version of these sequences is shown in the upper right. Sequence of each gene is from 5′ to 3′ from left to right and from top to bottom. Solid black lines separate one species' sequence from the next, and regions corresponding to sequences from exon 1 and exon 2 are shaded grey in the lower left half. Comparisons among species within the subgenus Sophophora are outlined in blue, whereas comparisons among species within the subgenus Drosophila are outlined in orange. The remaining black pixels indicate blocks of sequence similarity identified using LASTZ, as described in the Materials and Methods. The red and blue arrows indicate regions of collinear sequence similarity discussed in the main text.

To try to distinguish between these mechanisms, we performed pairwise comparisons of *yellow* genes and their 5′ intergenic sequences for all six species. As expected, significant sequence similarity was observed between homologous exons for all pairs of species (Figure 3). Outside of these regions, very little sequence similarity was observed for all but the most closely related pairs of species in each subgenus: *D. melanogaster* and *D. pseudoobscura* in the Sophophora subgenus, and *D. mojavensis* and *D. virilis* in the Drosophila subgenus. These two pairs of species provide the most power for investigating the molecular mechanisms responsible for interspecific differences in enhancer location. In both cases, one species in the pair has enhancer activity driving epidermal cell expression in the abdomen, thorax, and wing only in the 5′ intergenic region or only in the intron, whereas the other member of the pair has similar activities in both the 5′ intergenic region and the intron. Despite these differences in the genomic location of enhancers with similar tissue-specificity, we observed only collinear regions of sequence similarity (Figure 3, red and blue arrows). Such a pattern favors a model in which enhancers have been gained or lost through sequential sequence substitutions.

## Discussion

We found that the *cis*-regulatory architecture of *yellow* has changed repeatedly during the ∼40 million years since the six Drosophila species we examined last shared a common ancestor. This includes changes in the activity of homologous tissue-specific enhancers as well as changes in their relative genomic location. Sequence comparisons between the most closely related species examined showed no evidence of duplications or transpositions, suggesting that this diversity may have arisen through the gradual accumulation of sequence differences one (or a few) nucleotides at a time. As discussed below, these data provide insight into the independence of tissue-specific enhancers and the evolution of *cis*-regulatory architecture.

### Evolutionary conservation suggests interactions between tissue-specific enhancers

**Figure 4:**
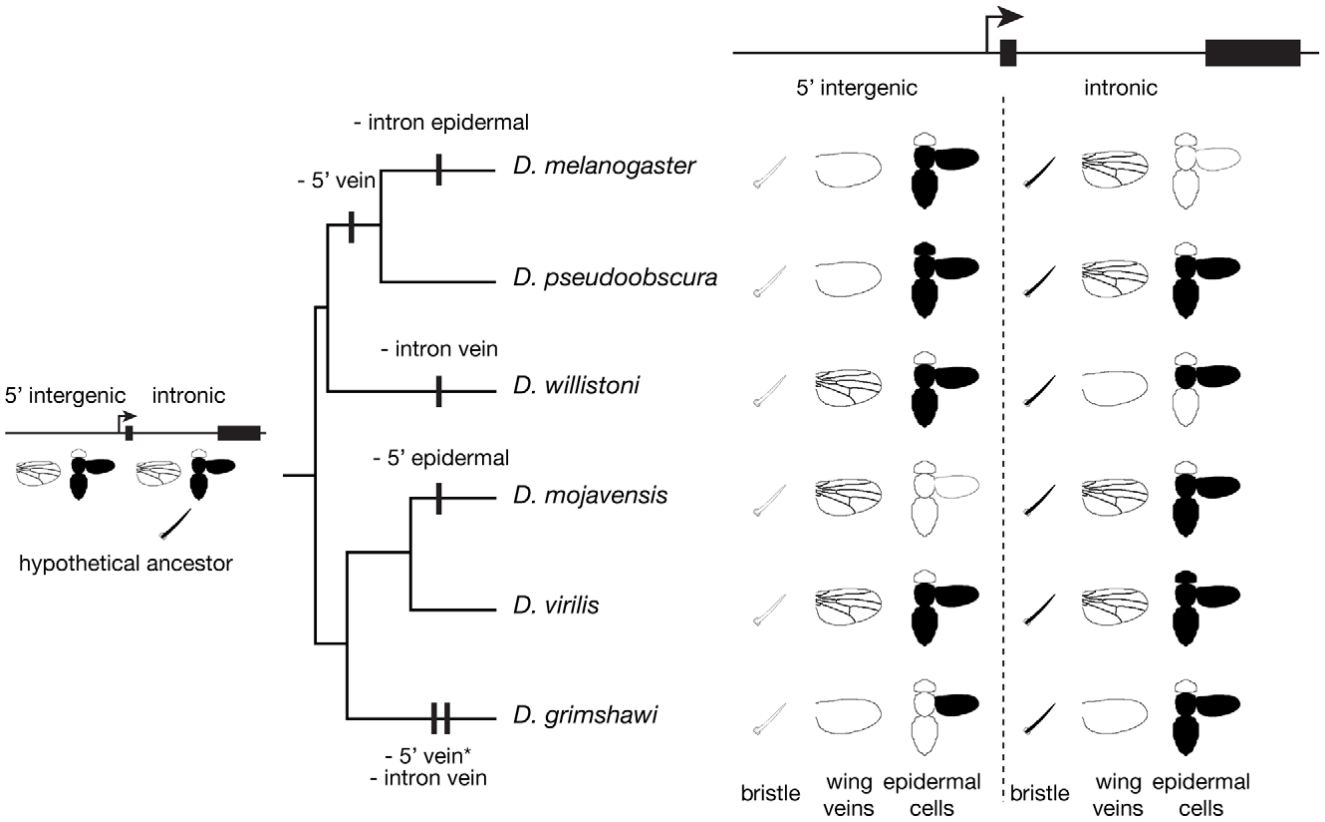
Dynamic *yellow cis*-regulatory architecture among Drosophila species. The schematic summarizes enhancer activity of 5′ intergenic and intronic sequences from each of the six species shown. In the bristle and epidermal cell schematics - the latter of which shows a head, thorax, abdomen, and wing - regions shaded in black showed GFP expression. For the wing vein schematics, pictures of wings including visible veins indicate vein enhancer activity. A phylogenetic tree showing the relationship among species is shown to the left of the enhancer expression summary. A hypothetical *cis*-regulatory architecture of the common ancestor of these six species is shown with wing vein and epidermal cell enhancers in both the 5′ intergenic and intronic regions. Vertical black bars on the branches of the phylogenetic tree indicate losses of enhancer activity. The asterisk next to “- 5′ vein activity” is because unpublished data from T. Werner and S.B. Carroll indicates that an enhancer driving expression in the wing veins (as well as additional wing epidermal cells) is located upstream of the 5′ intergenic region we examined; no information about the expression of this reporter gene in epidermal cells of the head, thorax, or abdomen was available.

Comparative studies that examine *cis*-regulatory sequences in an evolutionary context can uncover features overlooked by dissecting *cis*-regulatory sequences from a single species. For example, studies of *D. melanogaster yellow* identified non-overlapping DNA sequences that are necessary and sufficient to activate expression in epidermal cells of the body (i.e., abdomen and thorax) or wing, suggesting the presence of two distinct tissue-specific enhancers [12], [15], [16]. We found that these “wing” and “body” enhancer activities colocalize to the same genomic region in most species despite frequent evolutionary changes in the relative position of this region (Figure 4). This suggests that these enhancers are not fully independent, but rather interact in a way that constrains their evolution. For example, they might require close proximity to function properly at the native *yellow* locus because they share transcription factor binding sites and/or chromatin structure that promotes expression in pupal epidermal cells. Such colocalization was not observed for enhancers driving expression in bristle-associated cells or wing veins. Therefore, we propose that three evolutionarily independent enhancer modules regulate *yellow* expression: one controlling expression in bristle-associated cells, one controlling expression in the wing veins, and one controlling expression in the epidermal cells of the abdomen, thorax, head, and/or wing. Consistent with this proposal, a DNA fragment containing both the previously defined “body” and “wing” enhancers drives reporter gene expression in epidermal cells of the abdomen that is more representative of endogenous *D. melanogaster yellow* expression in those cells than that driven by a fragment containing the “body” enhancer alone [14].

### The genomic location of *yellow* enhancers has changed frequently during evolution

Examining divergent phenotypes in concert with a phylogenetic tree allows inferences to be made about the evolutionary changes that led to the observed trait diversity. To this end, Figure 4 shows the phylogenetic relationships among the species surveyed alongside a summary of the genomic locations of *yellow* enhancers from each species. Enhancer activity was considered present if reporter gene expression above background levels was observed in the tissue- or cell-type indicated regardless of the precise spatial pattern within that tissue.

To assess the evolutionary changes that gave rise to the observed diversity of *cis*-regulatory architecture, we must first infer the genomic locations of enhancers in the common ancestor of the six species studied. To do this, we considered each enhancer activity independently. The historical genomic location of bristle enhancer activity could be inferred with the most confidence: all six species showed bristle enhancer activity only in the intron, strongly suggesting that the common ancestor of these six species also had a bristle enhancer in this region. The ancestral locations of the wing vein and epidermal cell enhancers are less clear; these enhancer activities were found in the 5′ intergenic region, in the intron, and in both of these regions depending on the species surveyed. Furthermore, it is possible that there have been even more changes in *cis*-regulatory architecture than we were able to detect. For example, when functionally similar enhancers were observed in homologous genomic regions in different species, we made a conservative assumption that these enhancers were identical by descent. We also considered the possibility that *trans*-regulatory divergence might cause the activity of a heterologous enhancer to be different in *D. melanogaster* than it is in its native species (e.g., [12]); however, this is unlikely to explain the extensive changes in enhancer location we observed because of the very specific combination of *cis*- and *trans*-regulatory changes required to cause a spurious enhancer relocation with our assay.

Inferring the most likely genomic location(s) of wing vein and epidermal cell enhancers in the common ancestor requires an assumption about the relative likelihood of enhancer gain and enhancer loss in different lineages. Because mutations are expected to disrupt transcription factor binding sites more often than they are expected to create new ones, we assume that the loss of enhancer activity is more likely in all lineages than the gain of a novel tissue-specific enhancer. On the basis of this assumption, we propose that the most parsimonious explanation for the observed data is that the common ancestor had enhancers in both the 5′ intergenic and intronic regions of *yellow* that drove expression in the wing veins as well as in the abdomen, thorax, and wing epidermal cells. Such a scenario involves at least one loss of enhancer activity in the lineage leading to each of the species surveyed except *D. virilis*, as shown in Figure 4. While we find a common ancestor with redundant enhancers in the 5′ intergenic and intronic regions for both the wing veins and epidermal cells surprising, enhancers with overlapping tissue- and cell-type specific activities have been identified for other genes (e.g., [21]–[27]). For example, some genes are regulated by both primary and “shadow” enhancers that drive expression in the same cells [24]; the relative strength of these two enhancers may change over time. Scenarios involving a common ancestor with wing vein and/or epidermal cell enhancer activity in only one genomic region include multiple gains and losses in most lineages, which is presumably even less likely.

### Do changes in expression pattern and genomic location evolve together?

Regardless of the specific gains, losses, and/or relocations of *yellow* enhancers that occurred over the last 40 million years, it is clear that the genomic location of enhancer activities within and surrounding the *yellow* gene has changed multiple times. This finding is contrary to recent studies of seven genes expressed during embryogenesis that all have conserved genomic locations of enhancers between Drosophila and species that diverged over 100 million years ago [5], [7]. One way in which *yellow* differs from these genes is that its expression is much more divergent among species. This is presumably because *yellow* expression is required for pigmentation and pigmentation is a rapidly evolving trait among Drosophila species [28]. (See Text S1 for a discussion of how the observed changes in *yellow* enhancer activity relate to species-specific pigment patterns.)

Evolutionary processes resulting in divergent *yellow* expression might have allowed – or even facilitated – changes in the genomic location of its enhancers. For example, if changes in pigmentation are adaptive (or at least not maladaptive) mutations both inside and outside of existing *yellow* enhancers that affect its expression may not be eliminated by purifying selection, causing the gradual reorganization of enhancer architecture. *cis*-regulatory regions controlling conserved expression patterns, on the other hand, are more likely to have been subject to strong purifying selection, with new mutations that change enhancer activity and/or position selected against. Consistent with this proposal, we found that conserved expression of *yellow* in bristle-associated cells was controlled by an enhancer with a conserved genomic location, whereas divergent *yellow* expression in epidermal cells was controlled by enhancers with divergent locations (Figure 4). Divergent expression patterns are not a prerequisite for changing the location of *cis*-regulatory elements, however: the location of a *twist* enhancer with conserved activity has diverged between *D. melanogaster* and *D. virilis* [29], and changes in the genomic location of Polycomb/Trithorax response elements have also been observed between *Drosophila* species [30].

The prevalence of changes in enhancer position among species remains unknown. Many studies of *cis*-regulatory evolution have relied heavily on physical homology and sequence conservation to identify functionally homologous enhancers among species [31], creating an ascertainment bias that contributes to the prevailing view that enhancer position is usually conserved among species. Only once additional unbiased searches for enhancers using *in vivo* functional tests are performed will it be possible to determine whether nomadic enhancers are the exception or the norm.

## Materials and Methods

### Isolating *yellow* BAC clones

For five of the six species used in this study (*D. pseudoobscura*, *D. willistoni*, *D. mojavensis*, *D. virilis*, and *D. grimshawi*), BAC libraries (CHORI-222, DW_Ba, DM_CBa, DV_VBa and DG_Ba, respectively) were screened for clones containing *yellow* as well as its flanking genes. Nylon filters containing arrayed clones from the BAC libraries were obtained from BACPAC Resources (CHORI-222) and Arizona Genomics Institute (AGI) (DW_Ba, DM_CBa, DV_VBa and DG_Ba), and screened with [alpha-32-P]-labeled, random hexamer-primed probes synthesized using PCR amplicons from exons of the *yellow* gene; the *CG3777* gene, which is located 5′ of *yellow*; and either the *CG4165* (*D. mojavensis*) or *achete* (all other species) gene, both of which are located 3′ of *yellow*. (Primers and PCR conditions used to amplify the DNA template for each probe are available upon request.) Probe synthesis was performed as described in Molecular Cloning [32]. Unincorporated radionucleotides were removed using CentriSpin columns (Princeton Separations). Purified radioactive probes were denatured at 100°C for 5 minutes and placed on ice until they were added to the hybridization buffer containing the appropriate species specific BAC filter. BAC filter screening conditions and buffer recipes were as described in the AGI BAC Filter Manual available from the Arizona Genomics Institute (http://www2.genome.arizona.edu/research/protocols_bacmanual). After hybridizing each filter with a radioactive probe, the filter was washed and exposed to Kodak BioMax XAR films for 72 hours @ −80°C and developed.

Radiographs were used to identify clones as directed by the filter manufacturers (Arizona Genomics Institute and BACPAC Resources), and BACs that hybridized to all three probes were ordered. Upon receipt, each BAC clone was tested for the presence of *CG3777*, *yellow*, and *achete* or *CG4165* using PCR amplification. Table S1 lists all BAC clones found to contain *yellow* and at least one flanking gene. For *D. willistoni*, *D. mojavensis*, *D. virilis*, and *D. grimshawi*, BAC clones with code numbers 10L5, 4J24, 1A7 and 23K7, respectively, were used for reporter gene construction. For *D. melanogaster*, the RP98-13J2 BAC clone from the Roswell Park Cancer Institute Drosophila BAC Library, which was identified computationally and confirmed by PCR to contain *CG3777*, *yellow* and *achete*, was used for reporter gene construction. Note that none of the *D. pseudoobscura* BAC clones containing *yellow* had sufficient 5′ sequence to be used for reporter gene construction.

### Constructing reporter genes

For each species, 5′ intergenic and intronic regions of *yellow* were cloned into a plasmid containing piggyBac transposable element arms, a 3xP3-Enhanced Green Fluorescent Protein (EGFP) marker driving cytoplasmic GFP expression in the eyes [33], and a 300 bp attB site [20], [34] that we amplified from the pTA-attB plasmid provided by Michele Calos (Stanford University) and inserted into the unique XbaI site. As described in the main text, the 5′ end of the 5′ intergenic sequences was defined by the highly conserved region shown in Figure S1. The 5′ intergenic and intronic sequences from *D. melanogaster, D. subobscura*, *D. pseudoobcsura*, and the intron of *D. virilis yellow* were PCR amplified from BAC RP98-13J2, plasmid ysub-pBac [12], genomic DNA extracted from *D. pseudoobscura* (UCSD stock number 14011-0121.94), and plasmid yvir-pBac [10], respectively. Primer sequences used for these amplifications are available upon request. PCR products were ligated to the PCR 2.1 TOPO vector (Invitrogen), fully sequenced to identify clones with no PCR introduced mutations, and subcloned into the piggyBac-EGFP vector described above using the unique AscI restriction site.

For *D willistoni*, *D. mojavensis*, and *D. grimshawi*, both the 5′ intergenic and intronic regions, and for *D. virilis*, only the 5′ integenic region, were cloned into the piggyBac-EGFP vector using recombineering (http://recombineering.ncifcrf.gov/). Briefly, PCR was used to amplify 450–500 bp homology arms corresponding to the 5′ (left arm) and the 3′ (right arm) end of each target DNA sequence. PCR sewing was used to combine the left and right arms into a single fragment with a unique NheI restriction site between them. These DNA fragments were subcloned into PCR 2.1 TOPO, fully sequenced to identify clones without PCR introduced mutations, and subcloned into the piggyBac-EGFP vector using the unique AscI restriction site. Each piggyBac vector containing a species-specific pair of homology arms was linearized using the introduced NheI restriction site and electroporated into SW102 cells containing the *yellow* BAC from the appropriate species. Electroporation was conducted using Eppendorf Electroporator 2510 at 1250 Volts, with time constants ranging between 4.5–5. Following electroporation, SW102 cells were incubated in 1 ml LB at 30°C rotator for 1–1.5 hours, spread on LB agar plates supplemented with ampicillin (50 ug/ml), and grown overnight at 30°C to select for cells containing a circularized piggyBac-EGFP plasmid harboring the DNA of interest. Primers located in the piggyBac vector and in the target DNA sequences were paired to screen colonies for the existence and the direction of the DNA region of interest using PCR. Positive clones were confirmed by diagnostic digests using restriction enzymes specifically chosen for each construct, and the inserted DNA was completely sequenced to confirm once again that no experimentally introduced mutations were present. Next, a DNA fragment derived from pSLfa1180fa-nEGFP (Ernst Wimmer, Georg August University, Göttingen) containing an hsp70 promoter and the coding sequence for a nuclear EGFP protein was cloned into each piggyBac plasmid using the unique FseI restriction site. The resulting DNA transgene constructs were confirmed using appropriate diagnostic digests with restriction enzymes and sent to Genetics Services, Inc. (Cambridge, MA) where they were injected into the *w^−^*; attP-40 line of *D. melanogaster* [35]. This line contains a transgene expressing the ϕC31 site-specific integrase enzyme [34], which causes the targeted integration of each attB-containing piggyBac construct into the attP site on the *D. melanogaster* 2^nd^ chromosome. An “empty” piggyBac plasmid lacking any *yellow* sequence was also transformed into *D. melanogaster* and analyzed as a control to determine background levels of GFP expression.

### Analysis of reporter gene expression patterns

Homozygous transgenic *D. melanogaster* lines were obtained by crossing each transgenic *D. melanogaster* genotype to a 2^nd^ chromosome balancer line (w[*]; Kr[If-1]/CyO; D [*1*] */TM6B, Tb[+]*; Bloomington stock number 7197), intercrossing the F_1_ offspring, and then intercrossing selected homozygous F_2_ individuals. Homozygous transgenic animals were imaged at 70–80 hours APF, a stage which is recognized by pigmented wings as well as the presence of visible malpigian tubes on the anterior sides of the abdomen. The pupal case was removed prior to imaging using a probe and a pair of fine forceps.

To prepare the pupal bodies for confocal microscopy, the transparent pupal cuticle was kept in place without any tears and the pupa was mounted on a microscope slide with a drop of water and a coverslip. To prepare the pupal wings for confocal microscopy, the transparent pupal cuticle was removed and the whole fly was submerged in Milli-Q water. After the wings had unfolded, they were carefully detached from the rest of the pupa at the base of the wing where it connects to the thorax. Using a wide mouth pipette tip, each wing was transferred onto a microscope slide with a drop of water. A coverslip was applied and pressed gently to achieve full expansion of the wings. All specimens were imaged immediately after mounting using a Leica SP5 confocal microscope. Identical settings (e.g., laser power, pinhole size, etc) were used on the confocal microscope for all samples, and all raw confocal images of the same tissue (e.g., wings or bodies) were processed identically in Adobe Photoshop CS3. Results from the analysis of reporter genes containing 5′ intergenic and intronic sequences from *D. subobscura* are presented and discussed only in Figure S2 and its associated legend because the 5′ intergenic region surveyed in *D. subobscura* did not extend to the highly-conserved region used for all other species.

### Sequence analysis

*yellow* sequences and 5′ intergenic DNA from all species except *D. willistoni* were downloaded using the UCSC Genome Browser [36]. Specific assemblies and coordinates for each species were as follows: *D. melanogaster*, Apr. 2006 (BDGP R5/dm3) Assembly, chrX:246,727-255,037; *D. pseudoobscura*, FlyBase release r2.11, chrXL_group1e:4227884-4238281; *D. willistoni*, FlyBase release r1.3 scf2_1100000004909:5315142-5325379; *D. mojavensis*, Aug. 2005 (Agencourt prelim/droMoj2) Assembly, scaffold_6359:2,460,150-2,478,221; *D. virilis*, Aug 2005 (Agencourt prelim/droVir2) Assembly, scaffold_13042:3,903,783-3,920,981; *D. grimshawi*, Aug 2005 (Agencourt prelim/droGri1) Assembly, scaffold_24821:2,532,826-2,547,390. Homologous *D. willistoni* sequences were identified and downloaded using the BLAST implementation on FlyBase. These sequences were subject to repeat masking prior to analysis.

Alignments were performed using LASTZ (Release 1.02.00, built January 12, 2010), which was downloaded from Webb Miller's laboratory website (http://www.bx.psu.edu/). This unpublished software replaces the BLASTZ program developed by the same group [37]. Default settings were used except for the ” --mismatch = 2,23” option that sets an alternative threshold for the gap-free extension step. The basic structure of this analysis is as follows: all sequences 19 nucleotides long with matches in 12 specific positions were identified as “seeds”; seeds were extended in both directions without gaps until two mismatches were found in each end; extended seeds at least 23 nucleotides long were treated as “high scoring segment pairs” (HSPs); HSPs were converted into anchor points; anchor points were extended in both directions using gapped local alignments; and the coordinates of local alignments output by LASTZ were plotted using R statistical software [37]. The decision to allow a maximum of two mismatches during the gap-free extension stage was arbitrary, whereas the minimum length of extended seeds treated as HSPs (i.e., 23 nucleotides) was determined empirically by randomizing concatenated multi-species *yellow* sequences with the “Shuffle DNA” tool in the web-based “Sequence Manipulation Suite” [38] and iteratively testing length thresholds to find the smallest value that failed to identify any stretches of significant sequence similarity in the randomized sequence. Figure S3 shows the result of the same analysis with a decreased length threshold (”--mismatch-2,19”); 40 regions of significant sequence similarity were identified between the real and randomized sequences using these parameters.

## Supporting Information

Figure S1 Conserved region of non-coding sequence defines an orthologous endpoint for 5′ intergenic regions. (A) A schematic of the *yellow* gene is shown in yellow in which arrowheads point toward 3′ end of the gene, thicker yellow boxes indicate the protein coding sequences with the two exons, and narrower yellow boxes indicate the 5′ and 3′ UTRs. Below this image is a histogram representing the extent of sequence conservation among 12 *Drosophila* species, mosquito, honeybee, and beetle, as determined using a Multiz alignment [40] and phastCons Scores [41] and reported on the *D. melanogaster* UCSC Genome Browser ([42], http://genome.ucsc.edu/). The region shown is located on the X chromosome and extends from position 245,638 to 258,882 in the April 2006 (BDGP R5/dm3) assembly. Taller bars indicate greater sequence conservation. Below this histogram is a density plot indicating the amount of sequence conservation between each species and *D. melanogaster*; darker bars indicate higher degrees of conservation, as scored by phastCons [41]. Vertical green and blue lines in these density plots indicate a lack of collinearity with *D. melanogaster*. The red box indicates the conserved region used to determine an orthologous 5′ end to the intergenic fragments tested. (B) An alignment of sequences from the species examined in this study is shown for the boxed conserved region, which extends from positions 246,638 to 246,882 in the *D. melanogaster* genome (April 2006 (BDGP R5/dm3) assembly). Dashes indicate insertions or deletions among the twelve *Drosophila* species and honeybee sequence.
(0.57 MB PDF)

Figure S2 *D. subobscura* 5′ intergenic and intronic *yellow* sequences both contain epidermal cell enhancers. (A) A schematic of the *D. subobscura yellow* gene is shown with the amount of 5′ integenic (2.0 kb) and intronic (3.2 kb) DNA included in the reporter genes indicated. (B) Images of dorsal bodies (top row) and wings (bottom row) from an adult wild-type *D. subobscura* (left) and *D. melanogaster* transformant pupae carrying a GFP reporter gene controlled by sequences from the 5′ intergenic (middle) or intronic (right) region of *D. subobscura yellow* shown in (A). Like *D. pseudoobscura*, its closest relative among the species surveyed, expression in epidermal cells of the wing, abdomen, and thorax is driven by both the 5′ intergenic and intronic regions. Overall, the pattern of expression is similar between the two species, although some differences are apparent. For example, expression in the head cuticle is driven by intronic sequences from *D. subobscura*, but 5′ intergenic sequence from *D. pseudoobscura*; the 5′ intergenic region of *D. subobscura* drives expression in the wing veins whereas the *D. pseudoobscura* 5′ intergenic region does not; and the *D. subobscura* intron lacks the elevated spot of expression in the anterior part of the wing seen in *D. pseudoobscura*.
(0.10 MB PDF)

Figure S3 Alternative sequence alignment parameters also show primarily collinear sequence similarity. Relaxing alignment parameters identified more regions of sequence similarity between species, but still showed no evidence of large duplications or transpositions. Figure format is as described in the legend to Figure 3 in the main text, and analysis conditions are as described in the Materials and Methods.
(0.46 MB PDF)

Table S1 BAC clones containing *yellow* and flanking genes.
(0.04 MB PDF)

Text S1 Supporting text.
(0.05 MB DOC)
