# Supplementary material for: Nomadic Enhancers: Tissue-Specific cis-Regulatory Elements of yellow Have Divergent Genomic Positions among Drosophila Species
Source: PLoS Genet. 2010 Nov 24;6(11):e1001222. doi: 10.1371/journal.pgen.1001222 (PMC2996884; doi:10.1371/journal.pgen.1001222)
Supplement: Figure S1 — Conserved region of non-coding sequence defines an orthologous endpoint for 5′ intergenic regions. (A) A schematic of the yellow gene is shown in yellow in which arrowheads point toward 3′ end of the gene, thicker yellow boxes indicate the protein coding sequences with the two exons, and narrower yellow boxes indicate the 5′ and 3′ UTRs. Below this image is a histogram representing the extent of sequence conservation among 12 Drosophila species, mosquito, honeybee, and beetle, as determined using a Multiz alignment [40] and phastCons Scores [41] and reported on the D. melanogaster UCSC Genome Browser ([42], http://genome.ucsc.edu/). The region shown is located on the X chromosome and extends from position 245,638 to 258,882 in the April 2006 (BDGP R5/dm3) assembly. Taller bars indicate greater sequence conservation. Below this histogram is a density plot indicating the amount of sequence conservation between each species and D. melanogaster; darker bars indicate higher degrees of conservation, as scored by phastCons [41]. Vertical green and blue lines in these density plots indicate a lack of collinearity with D. melanogaster. The red box indicates the conserved region used to determine an orthologous 5′ end to the intergenic fragments tested. (B) An alignment of sequences from the species examined in this study is shown for the boxed conserved region, which extends from positions 246,638 to 246,882 in the D. melanogaster genome (April 2006 (BDGP R5/dm3) assembly). Dashes indicate insertions or deletions among the twelve Drosophila species and honeybee sequence. (0.57 MB PDF) [file pgen.1001222.s001.pdf]

Supplementary Figure 1
Kalay and Wittkopp

A

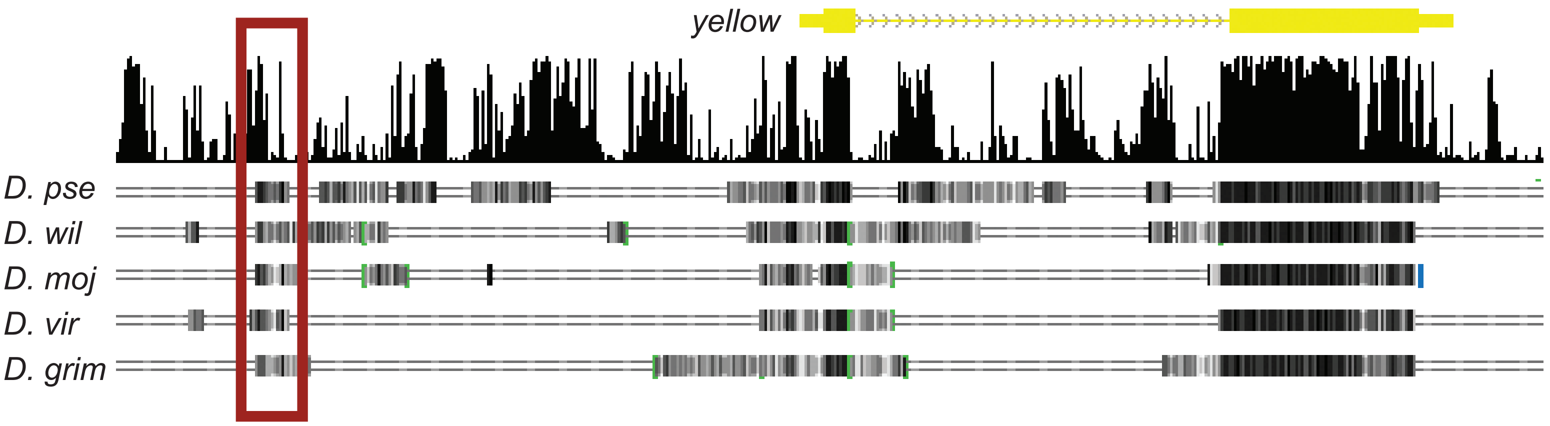


B

```
    D. melanogaster  acagtcggtttgtagttgc-gatttaaatagtttttaattaataactaattgcagcgaga-------cga
  D. pseudoobscura  --------ttgacagttgc--atttaaatagtttttaattgaaaactaattgccacgagt-------gga
     D. willistoni  ---------------tttc-agtgcaattagttttcaattaaaaactatttaaatcgcaaccgattccga
     D. mojavensis  acaactcgttagcagctgctgatttaaatagtttttaattgccagcaaattg-agccgaa-------taa
        D. virilis  ccaactcgttagcagctggcgatttaaatagtttttaattggcagctaattg-agctgaa-------tag
      D. grimshawi  ----------------------tttaaatagtatttgtatggaaactaattg-agctgaa-------cga

   D. melanogaster  gtgcca-----------aa----acaagaca-g----tggtcgaca-------------tagctgtga-c
  D. pseudoobscura  gtgcca-----------aa----gcgcgacg-acagctggacgaca-------------tggttgtga-c
     D. willistoni  ttgccagtcagactcccaa----gcaatccg-g----caaacgccacagaaactataactgaaaatga-a
     D. mojavensis  gtgcca-----------aa----actggacaag----tgcgtggca-----------gctggccgaca-c
        D. virilis  ctgcca-----------aa----actggacaag----tgcgtggca-----------actggccgaca-c
      D. grimshawi  gttcca-----------aacaggaaagaaaatg----tacgcgata-----------------catcagc

   D. melanogaster  gttttcaatgcag------caacatggtaacatggcaactat-tt------------------------a
  D. pseudoobscura  gtttccagctctc------------------gctgcaacgctctt------------------------a
     D. willistoni  aactcaaaaacaa---aaccaaactagttaaatgcgaaatat-ttctctcttgggtttcatttccttcac
     D. mojavensis  gtcttgtcgtctc------t--------------------------------------------------
        D. virilis  gtcttgtcgtctc------tta---------agccaaacagt-gt----------------------ggg
      D. grimshawi  atcttatcgtctc------tcgt-tatttgtattcaagccac-tt----------------------aaa

   D. melanogaster  gcca-gtt------------aattggcaa--caa-gccgacagcgatatt----------------agca
  D. pseudoobscura  gccacttt------------aattggcag--cag---cagcagcagccaa----------------aaca
     D. willistoni  gcca-tttggaaattcttggaattgcata--caa--ttggcagaaataat----------------aata
     D. mojavensis  ------------------------------------------------ca---------------cacaa
        D. virilis  gcac-ac---------------acaacaa--cagcaacaacaacaatcca---------------cacaa
      D. grimshawi  tgcc-acc------------agctgccaa--taccaccggcacccatccg--------------ttgctg

   D. melanogaster  gcagcaacaacagcttt--tcaccaggaaatcaata-----atagtttgtccaaaa--cata------at
  D. pseudoobscura  gcggcaacaacaacttt--tcaccaggaaatcggca-----a---atgatccgaaagccaaacgagtcgc
     D. willistoni  ataataataataattatgagcataaataagtagata-----gtaaaggaaacaaat--aaaa------ct
     D. mojavensis  gagaca---gcaacttt--tcaccaggaaatcgaga-----a----------aacattcaaa------aa
        D. virilis  ccaacaatgacaacttt--tcaccaggaaaccaagaaaaaca----------aaaatacaaa------at
      D. grimshawi  gcagtactgacaacttt--tcaccaggaagccgaca-----a----------gaaaacaaaa------aa

   D. melanogaster  aataaaataaagccg--
  D. pseudoobscura  aataaccgaaagctg--
     D. willistoni  gacacacacacacag--
     D. mojavensis  caaaaaca---------
        D. virilis  caaaaaaa---------
      D. grimshawi  aagagact-----tgca
```
