# Supplementary figures and images for: Nomadic Enhancers: Tissue-Specific cis-Regulatory Elements of yellow Have Divergent Genomic Positions among Drosophila Species
Source: PLoS Genet. 2010 Nov 24;6(11):e1001222. doi: 10.1371/journal.pgen.1001222 (PMC2996884; doi:10.1371/journal.pgen.1001222)

Supplementary Figure 2  
Kalay and Wittkopp

A *D. subobscura* yellow

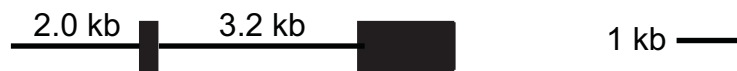

B

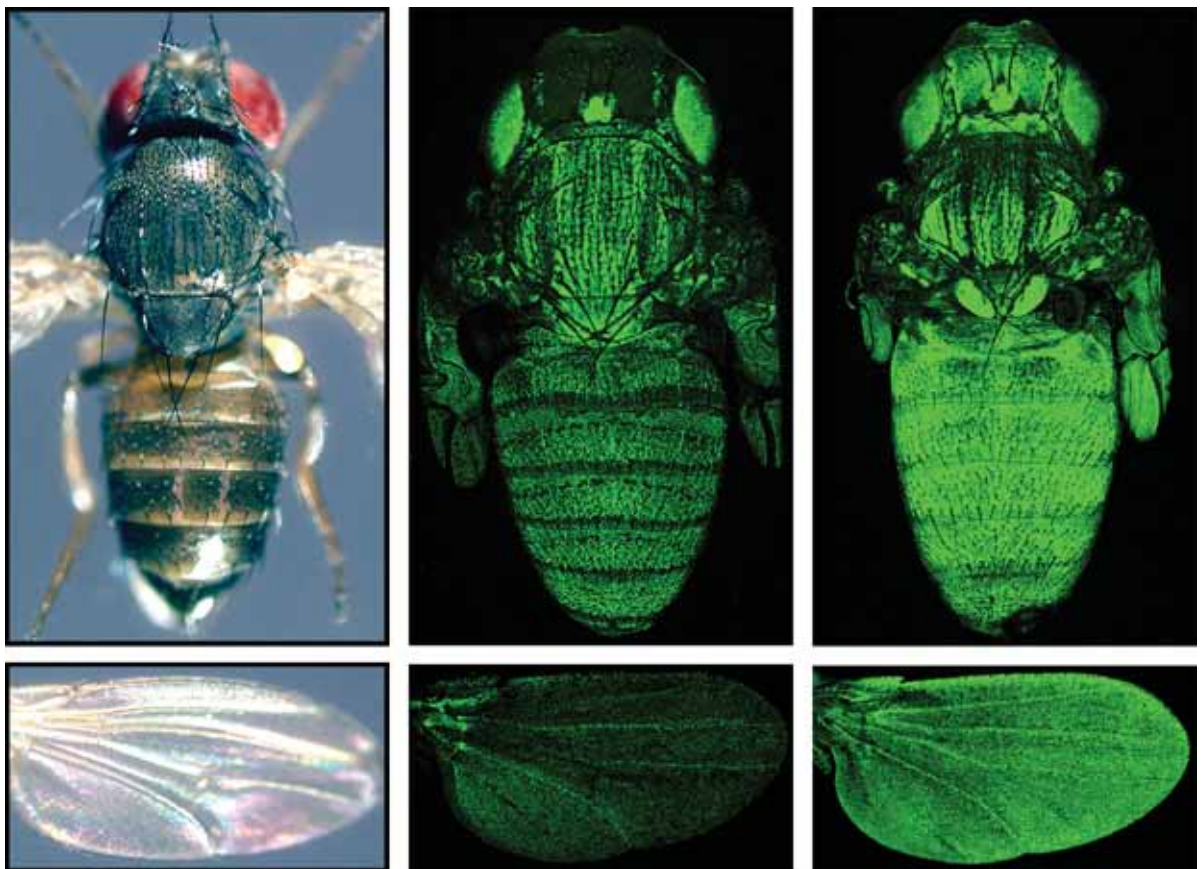

Supplement: Figure S2 — D. subobscura 5′ intergenic and intronic yellow sequences both contain epidermal cell enhancers. (A) A schematic of the D. subobscura yellow gene is shown with the amount of 5′ integenic (2.0 kb) and intronic (3.2 kb) DNA included in the reporter genes indicated. (B) Images of dorsal bodies (top row) and wings (bottom row) from an adult wild-type D. subobscura (left) and D. melanogaster transformant pupae carrying a GFP reporter gene controlled by sequences from the 5′ intergenic (middle) or intronic (right) region of D. subobscura yellow shown in (A). Like D. pseudoobscura, its closest relative among the species surveyed, expression in epidermal cells of the wing, abdomen, and thorax is driven by both the 5′ intergenic and intronic regions. Overall, the pattern of expression is similar between the two species, although some differences are apparent. For example, expression in the head cuticle is driven by intronic sequences from D. subobscura, but 5′ intergenic sequence from D. pseudoobscura; the 5′ intergenic region of D. subobscura drives expression in the wing veins whereas the D. pseudoobscura 5′ intergenic region does not; and the D. subobscura intron lacks the elevated spot of expression in the anterior part of the wing seen in D. pseudoobscura. (0.10 MB PDF) [file pgen.1001222.s002.pdf]

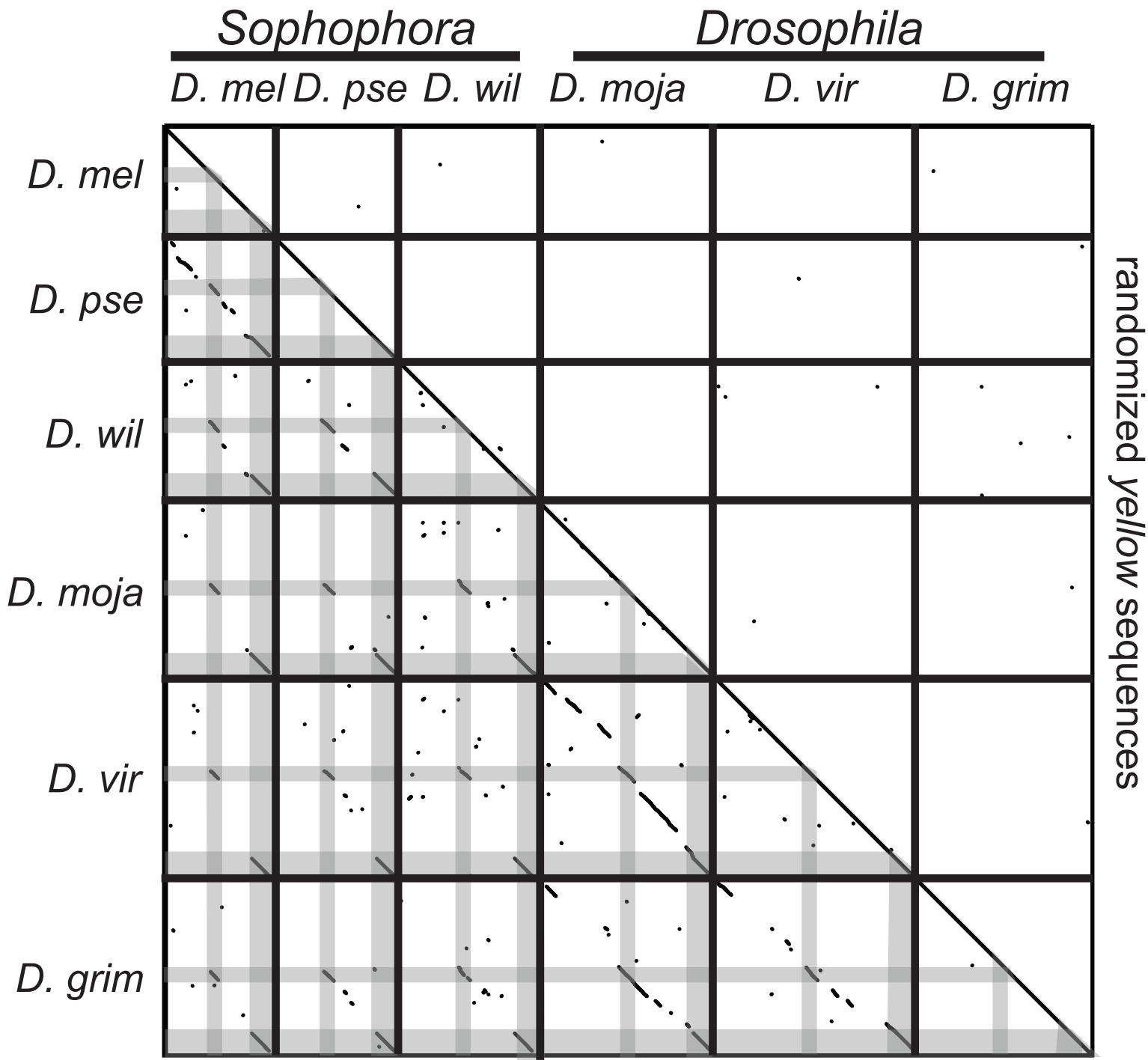

Supplement: Figure S3 — Alternative sequence alignment parameters also show primarily collinear sequence similarity. Relaxing alignment parameters identified more regions of sequence similarity between species, but still showed no evidence of large duplications or transpositions. Figure format is as described in the legend to Figure 3 in the main text, and analysis conditions are as described in the Materials and Methods. (0.46 MB PDF) [file pgen.1001222.s003.pdf]
