# Supplementary material for: Nomadic Enhancers: Tissue-Specific cis-Regulatory Elements of yellow Have Divergent Genomic Positions among Drosophila Species
Source: PLoS Genet. 2010 Nov 24;6(11):e1001222. doi: 10.1371/journal.pgen.1001222 (PMC2996884; doi:10.1371/journal.pgen.1001222)
Supplement: Table S1 — BAC clones containing yellow and flanking genes. (0.04 MB PDF) [file pgen.1001222.s004.pdf]

Supplementary Table 1: BAC clones containing *yellow* and flanking genes

Species	BAC clone number[1]	Sequences included in the BAC clone				
		CG3777	consv[2]	*yellow*	*achete*	*CG4165*
D. pseudoobscura	CH222-46B19	-	NA[3]	+	-	NA
	CH222-11G23	-	NA	+	-	NA
	CH222-11H21	-	NA	+	-	NA
D. willistoni	DW18M19	-	NA	-	-	NA
	DW14D14	-	NA	+	+	NA
	**DW10L5**	+	+	+	+	NA
D. mojavensis	DM32A21	+	+	+	NA	+
	DM14M10	+	+	+	NA	+
	**DM4J24**	+	+	+	NA	+
	DM1M5	+	+	+	NA	-
	DM1C16	+	NA	-	NA	-
	DM9D16	+	+	+	NA	+
	DM2K5	+	NA	-	NA	-
	DM31I1	+	NA	-	NA	-
	DM9O22	+	+	+	NA	-
D. virilis	DV98O14	-	+	+	+	NA
	DV106J1	-	+	+	+	NA
	**DV1A7**	-	+	+	+	NA
	DV50B1	-	+	+	+	NA
	DV2K10	-	+	+	+	NA
	DV30O8	-	+	+	+	NA
	DV88F9	-	+	+	+	NA
	DV22J8	-	+	+	+	NA
	DV52F20	-	-	+	+	NA
	DV124G7	-	-	+	+	NA
	DV20J7	-	+	+	+	NA
	DV136F3	-	+	+	+	NA
D. grimshawi	DG8L1	+	+	+	+	NA
	DG18J1	+	+	+	+	NA
	DG27E24	+	+	+	+	NA
	DG41C3	+	+	+	+	NA
	DG3B4	+	+	+	+	NA
	DG3M22	+	+	+	+	NA
	DG8J4	-	NA	+	+	NA
	DG38N3	-	NA	+	+	NA
	DG4G9	-	NA	-	-	NA
	**DG23K7**	+	+	+	+	NA

[1] BAC clones used for reporter gene construction are shown in bold

[2] conserved region of sequence 5' of *yellow* shown in Supplementary Figure 1

[3] NA=Not Available (ie., untested)
