# Supplementary material for: Nomadic Enhancers: Tissue-Specific cis-Regulatory Elements of yellow Have Divergent Genomic Positions among Drosophila Species
Source: PLoS Genet. 2010 Nov 24;6(11):e1001222. doi: 10.1371/journal.pgen.1001222 (PMC2996884; doi:10.1371/journal.pgen.1001222)
Supplement: Text S1 — Supporting text. (0.05 MB DOC) [file pgen.1001222.s005.doc]

**Supporting Text**

***yellow* enhancer activity often, but not always, evolves with pigmentation**

In *Drosophila*, *cis*-regulatory changes affecting *yellow* expression often correlate with changes in pigmentation among species [1-5], suggesting that they have contributed to the evolution of this trait. Indeed, we observed a correlation between pigmentation and enhancer activity in most of our dataset; however, not all pigment patterns were reflected in reporter gene expression. For example, pigment spots on the body of *D. mojavensis* and on the wings of *D. grimshawi* were not observed in the expression pattern of either of the reporter genes from these species. This could be because these particular pigment patterns are controlled by another pigmentation gene such as *tan* [6-8]. Alternatively, enhancers driving *yellow* expression in these patterns may be located outside of the regions surveyed; an additional wing enhancer was found in *D. grimshawi* 5’ of intergenic region we tested (T. Werner and S.B. Carroll, personal communication). Finally, *trans*-acting factors controlling *yellow* expression may have diverged between *D. melanogaster* and *D. mojavensis* or *D. grimshawi* such that sequences drive expression in a different pattern when inserted into the *D. melanogaster* genome than they do in their native species. Such *trans*-regulatory changes are known to exist between *D. melanogaster* and *D. virilis* [1] and between *D. melanogaster* and *D. guttifera* [5].

In addition to pigment patterns not reflected in reporter gene expression, we also observed reporter gene expression not reflected in pigment patterns. Intronic sequences from *D. pseudoobscura* activated reporter gene expression in an anterior region of *D. melanogaster* wings despite the fact that adult *D. pseudoobscura* lack pigmentation in this area (Figure 2F, arrowhead). This expression pattern does not appear to be an artifact of the heterologous transgenic host because a similar pattern is seen in native *D. pseudoobscura* Yellow expression (see Figure 1 in [2]). Interestingly, *D. tristis*, which is a member of the obscura group to which *D. pseudoobscura* also belongs, has a similar pattern of *yellow* expression in pupal wings controlled by an intronic enhancer and does display a corresponding spot of pigmentation on its adult wings [3]. This spot of wing pigmentation appears to be a derived trait in the obscura group [3], thus the presence of this expression pattern in *D. pseudoobscura* suggests that the novel *yellow* enhancer activity in this wing spot preceded other changes, such as a coincident decrease in Ebony protein expression [2,9], that are also required for wing spot formation.

**References for Supporting Information**

1. Wittkopp PJ, Vaccaro K, Carroll SB (2002) Evolution of yellow gene regulation and pigmentation in Drosophila. Curr Biol 12: 1547-1556.

2. Gompel N, Prud'homme B, Wittkopp PJ, Kassner VA, Carroll SB (2005) Chance caught on the wing: cis-regulatory evolution and the origin of pigment patterns in Drosophila. Nature 433: 481-487.

3. Prud'homme B, Gompel N, Rokas A, Kassner VA, Williams TM, et al. (2006) Repeated morphological evolution through cis-regulatory changes in a pleiotropic gene. Nature 440: 1050-1053.

4. Jeong S, Rokas A, Carroll SB (2006) Regulation of body pigmentation by the Abdominal-B Hox protein and its gain and loss in Drosophila evolution. Cell 125: 1387-1399.

5. Werner T, Koshikawa S, Williams TM, Carroll SB (2010) Generation of a novel wing colour pattern by the Wingless morphogen. Nature 464: 1143-1148.

6. Jeong S, Rebeiz M, Andolfatto P, Werner T, True J, et al. (2008) The evolution of gene regulation underlies a morphological difference between two Drosophila sister species. Cell 132: 783-793.

7. True JR, Yeh SD, Hovemann BT, Kemme T, Meinertzhagen IA, et al. (2005) Drosophila tan encodes a novel hydrolase required in pigmentation and vision. PLoS Genet 1: e63.

8. Wittkopp PJ, Stewart EE, Arnold LL, Neidert AH, Haerum BK, et al. (2009) Intraspecific polymorphism to interspecific divergence: genetics of pigmentation in Drosophila. Science 326: 540-544.

9. Wittkopp PJ, True JR, Carroll SB (2002) Reciprocal functions of the Drosophila yellow and ebony proteins in the development and evolution of pigment patterns. Development 129: 1849-1858.
